# Effectiveness and Safety of Anti-Tumor Necrosis Factor-Alpha Agents Treatment in Behcets’ Disease-Associated Uveitis: A Systematic Review and Meta-Analysis

**DOI:** 10.3389/fphar.2020.00941

**Published:** 2020-06-24

**Authors:** Yunwei Hu, Zhaohao Huang, Shizhao Yang, Xiaoqing Chen, Wenru Su, Dan Liang

**Affiliations:** State Key Laboratory of Ophthalmology, Zhongshan Ophthalmic Center, Sun Yat-sen University, Guangzhou, China

**Keywords:** anti-TNF-α, Behcets’ disease-associated uveitis, inflammation remission rate, visual acuity improvement, central macular thickness decrease, corticosteroid-sparing effect, adverse events, meta-analysis

## Abstract

**Purpose:**

We conducted a systematic review and meta-analysis to determine the effectiveness and safety of anti-tumor necrosis factor-alpha (TNF-α) agents in the treatment of Behcets’ disease (BD)-associated uveitis.

**Method:**

Three electronic databases, Embase, MEDLINE, and the Cochrane Library, were searched for eligible papers focusing on the anti-TNF-α agents treatment in BD-associated uveitis with at least 6 months follow-up time. A systematic review and meta-analysis was conducted on selected papers with appropriate clinical and methodological homogeneity. The effectiveness outcomes included inflammation remission, visual acuity (VA) improvement, central macular thickness (CMT) decrease, corticosteroid (CS)-sparing effects, and the safety outcomes included minor and severe drug-related adverse events (AEs).

**Result:**

From Jan 2010 to Dec 2019, there were 504 records produced in total, in which 18 clinical trials were selected for meta-analysis (15 trials were retrospective studies, and 3 were prospective studies). The number of patients in each study ranged from 11 to 163 and the mean follow-up time from 0.9 to 6.44 years. During the follow-up, the pooled inflammation remission rate was 68% with a 95% confidence interval (CI) of 0.59–0.79, VA improvement rate was 60% (95% CI 0.47–0.77), CMT decrease was 112.70 μm (95% CI 72.8–153.0 μm). The proportions of patients who had CS-suspended and CS-tapered reached 38% (95% CI 0.23–0.65) and 34% (95% CI 0.16–0.70), respectively. The severe AEs were reported but not common, which included severe infusion reactions, pneumonia, bacteremia, tuberculosis, melanoma, and lymphoma.

**Conclusion:**

Anti-TNF-α agents treatment has high effectiveness including efficient inflammation remission, satisfactory VA improvement, obvious CMT reduction, and significant CS-sparing effects. Although some drug-related AEs were reported, the incidence of severe AEs was acceptable. Anti-TNF-α agents treatment is a promising option for controlling BD-associated uveitis.

## Introduction

Behçets’ disease (BD) is a rare and idiopathic multisystem inflammatory disorder with diverse clinical manifestations, including recurrent oral and genital ulcers, cutaneous vascular lesions, central nervous system, and ocular impairment (Hatemi et al., 2013; Takeuchi et al., 2015; Zeidan et al., 2016). The incidence of BD varies according to geographical location, with high prevalence along the ancient silk road (15–420 cases per 100,000 population) and very low prevalence in North America (0.12–0.33 cases per 100,000 population) and in Western countries (Cho et al., 2012; Pineton de Chambrun et al., 2012).

Ocular involvement occurs in 30–70% of cases of BD (Greco et al., 2018) and usually presents as chronic, relapsing uveitis or retinal vasculitis, which result in severe complications, such as intractable macular edema, retinal vessels obstruction, nerve fibers atrophy, and opacity of optical medium. About 25% of patients of BD-associated uveitis become blind as a result of irreversible vision impairment after multiple recurrences. It is urgent to control the inflammation and reduce the remission with effective therapy (Kitaichi et al., 2007; Kacmaz et al., 2008; Greco et al., 2018).

It is recommended to use systematic corticosteroids (CSs) together with immunosuppressive agents, such as azathioprine (AZA), cyclosporin (CsA), and methotrexate (MTX) for the treatment of BD-associated uveitis (Hatemi et al., 2018; Leccese et al., 2019). Despite successfully decreasing inflammation in some BD-associated uveitis, the combined treatment often fails to prevent relapses in a majority of those patients. What’s more, local and systemic side effects occur when large-dose steroids are used, and relapses are frequently seen after discontinuation of steroids in refractory patients (Kaklamani and Kaklamanis, 2001; Levy-Clarke et al., 2014). New treatment strategies are urgently needed.

Biological therapies, such as the inhibition of TNF-α, have emerged as preferred treatment alternatives for treatment (Levy-Clarke et al., 2014). As we all know that TNF-α is the major pre-inflammation factor involving autoimmune diseases. Anti-TNF-α agents are recommended as a first- or second-line therapy or even as a rescue therapy after the failure of conventional drugs in chronic noninfectious uveitis with diverse etiologies (Levy-Clarke et al., 2014). Many previous studies have reported the efficacy and safety of anti-TNF-α agents, such as infliximab (IFX) and adalimumab (ADA) in chronic noninfectious uveitis (Sfikakis et al., 2004; Biester et al., 2007; Dhingra et al., 2009; Martel et al., 2012; Suhler et al., 2013; Mercier et al., 2018). Recently, two Randomized Controlled Trials (RCTs), VISUAL I (Jaffe et al., 2016) and VISUAL II (Nguyen et al., 2016) have been conducted to evaluate the therapy of adalimumab in intermediate, posterior and pan-uveitis related different etiologies. What’s more, ADA was approved in non-infectious uveitis by US FDA^1^ and European Medicines Agency (EMA)^2^ in 2016, also approved by China National Medical Products Administration (NMPA) in 2020.

However, the heterogeneity of drug response is still existed among different types of uveitis and the prognosis of BD-associated uveitis is usually worse than other etiological uveitis. As we know, until now there is few RCT or systematic review evaluating the efficacy and safety of anti-TNF-α agents specifically on BD-related uveitis, so it is needed to get more evidence for ophthalmologists to make right treatment decision. Based on the above consideration, we performed a systematic literature review and meta-analysis to summarize the currently available evidence regarding the effectiveness and safety of anti-TNF-α therapy in the treatment of BD-associated uveitis.

## Methods

A systematic review and meta-analysis was conducted to integrate information presented in studies on the effectiveness and safety of anti-TNF-α therapy in the treatment of uveitis associated with BD published in the past 10 years. This review was carried out following the preferred reporting items for systematic review and meta-analysis (PRISMA) statements and the protocol followed the PRISMA-Protocol guidelines (Preferred reporting items for systematic review and meta-analysis protocols (PRISMA-P) 2015: elaboration and explanation, 2016).

### Search Strategy

We searched the electronic Medline, Embase, and Cochrane Library databases for papers published from January 2010 to September 2019. The search was restricted in the abstract\keywords\title fields to cut down on irrelevant literature. The index words were “Behcet” or “Behcet’s” or “Behcet disease” or “Behcet’ disease” or “Behcet’s disease” crossed with “anti tumor necrosis factor alpha” or “anti TNF alpha” or “TNF alpha inhibitors” or “tumor necrosis factor alpha inhibitors” or “TNF-α inhibitors” or “anti-TNF-α” or “adalimumab” or “infliximab”. Ocular involvement was searched by “uveitis” or “iridocyclitis” or “retinitis” or “retinal vasculitis” or “panuveitis”. Prospective open-label trials, uncontrolled case series reports and summaries of conferences were included to provide more evidence.

### Inclusion Criteria

The inclusion criteria were defined as follows: 1) BD-associated uveitis studies or studies including data of BD-associated uveitis which could be extracted separately, 2) patients received anti-TNF-α therapy, 3) the mean follow-up time was at least 6 months, and 4) at least 10 patients were included in the study to avoid bias.

It is generally recommended anti-TNF-α agents to be used for more than 6 months in patients of chronic non-infectious uveitis. What’s more, in most studies, the examinations and evaluations were done every 6 months during follow-up time.

### Study Selection and Quality Evaluation

Two independent authors (YH and WS) identified the relevant studies according to the inclusion criteria. The quality and risk of bias of each trial was evaluated using the Newcastle-Ottawa Scale (Wells et al., 2009). Any disagreements were resolved by discussion and, if required, referred to a third reviewer.

### Outcome Measures

The effectiveness outcome measures included the proportion of patients with inflammation remission, visual acuity (VA) improvement, central macular thickness (CMT) decrease and CS-sparing effects.

The safety outcome measures were the number and severity of adverse events (AEs). AEs included, but were not limited: 1) new-onset or reactivated infections, 2) gastrointestinal discomfort, 3) injection site or allergic reactions, 4) immunogenicity-related AEs.

### Data Extraction and Analysis

The evaluation of outcomes was performed for each patient. Descriptions of dichotomous outcome were graded dichotomously by “yes” or “no” responses. We retrieved the total number of included patients and the number who experienced the outcome. We reported all proportions of patients who experienced outcomes and related 95% confidence intervals (95% CIs). Dichotomous data were preferentially reported in this review as risk ratios using a Mantel-Haenszel fixed-effects (FE) model and 95% CIs. The pooled prevalence rates and their 95% CIs were estimated using a random or fixed effects model. For continuous outcomes, we retrieved the mean and standard deviation (SD). The pooled mean and SD were estimated using a random or fixed effects model. A random-effects model was used if the statistical heterogeneity > 50% and p < 0.1. Otherwise, a fixed-effects model was used.

All analyses were performed in STATA 12. Meta-analysis was utilized to identify the pooled rates of inflammation remission, VA improvement, CMT decrease, and CS-sparing effects. Safety outcomes were analyzed by qualitative synthesis with a focus on the description instead of meta-analysis. Subgroup analyses were conducted to explore the sources of between-study heterogeneity. The subgroups were divided by study location, study design, the duration of follow-up time and the choice of anti-TNF-α agents. Potential publication bias was assessed by Egger’s test and presented in funnel plots.

## Results

### Study Selection and Characteristics

**Figure 1:**
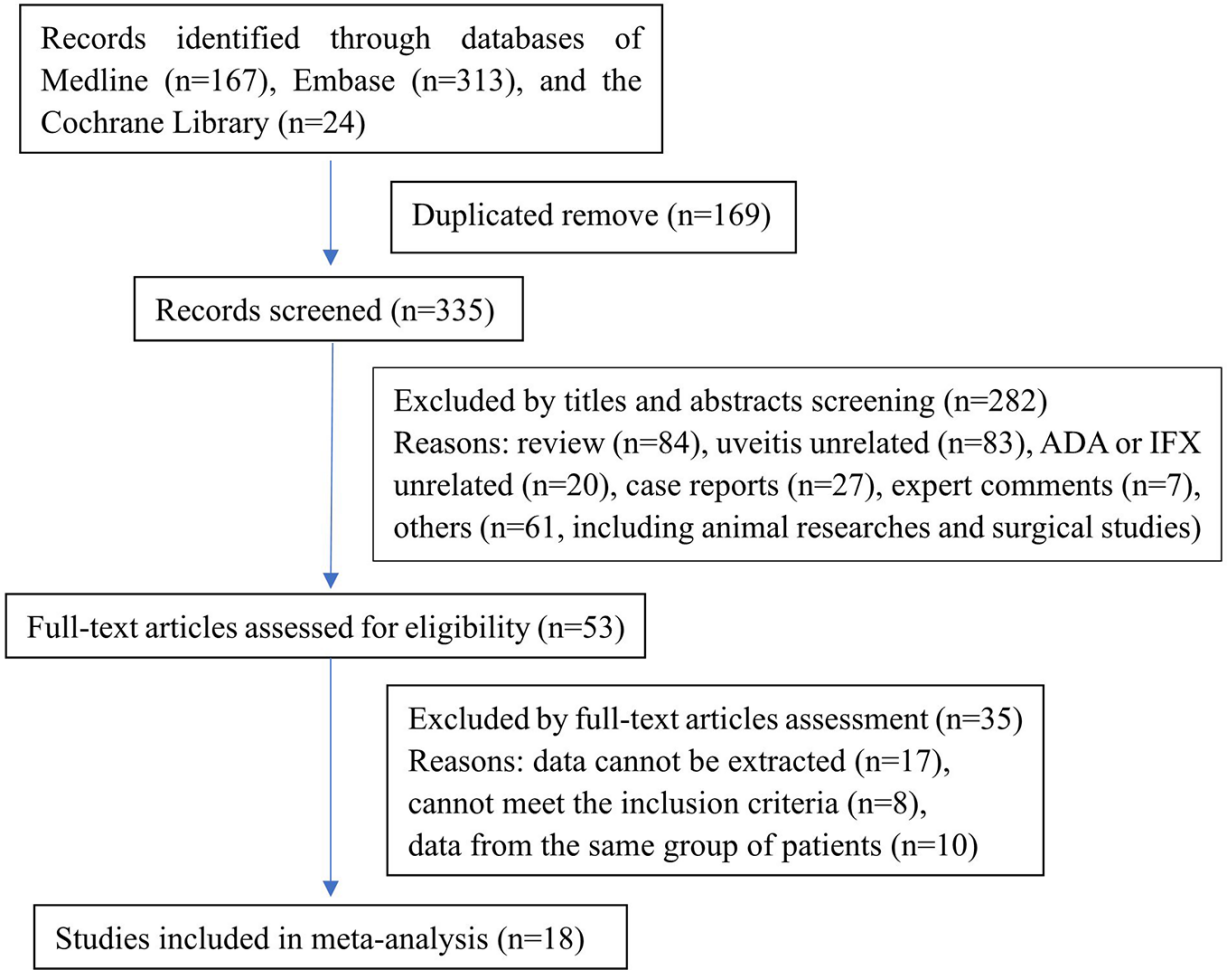
Flow diagram demonstrating the study selection process. ADA, adalimumab; IFX, infliximab.

The search of literature produced 504 records (MEDLINE: 167, Embase: 313, CENTRAL: 24). No additional records were identified through other sources or manual searches. After duplicate removal and title and abstract screening, 53 articles were identified and examined in detail. Finally, we included 18 articles in the meta-analysis. A flow diagram demonstrated the process of study selection (**Figure 1**). Most studies (15/18) were retrospective, and only three studies were prospective. The anti-TNF-α agents researched in these studies included adalimumab and infliximab. The quality evaluation of the included studies was done according to the Newcastle-Ottawa Scale.

The characteristics of these studies (Bawazeer et al., 2010; Keino et al., 2011; Cantini et al., 2012; Okada et al., 2012; Al Rashidi et al., 2013; Bejerano et al., 2013; Calvo-Río et al., 2013; Hamuryudan et al., 2013; Mesquida et al., 2013; Calvo-Río et al., 2014; Interlandi et al., 2014; Takeuchi et al., 2014; Domínguez Casas et al., 2017; Fabiani et al., 2017; Degirmenci et al., 2018; El Garf et al., 2018; Martín-Varillas et al., 2018; Katsuyama et al., 2019; Yalcindag and Kose, 2019) were described in **Table 1** and **Supplementary Table 1**. The 18 articles comprised 968 patients in total and the number of patients in each study ranged from 11 to 164. Excluding one study which enrolled 20 patients did not give sex information, 64.66% (613/948) of patients in other 17 studies were male. The mean ages ranged from 24.3 to 45.1 years and the mean follow-up time ranged from 0.9 to 6.44 years.

**Table 1:** Study characteristics of the included studies.

First author (year)	Location	Continent	Study design	Anti-TNF-α agents	Sample size	Male (%)	Mean age (SD)	Remission (%)^a^	Follow-up (years)^b^	Quality score^c^
Al Rashidi et al. (2013)	Saudi Arabia	Asia	Retrospective study	IFX	19	18 (94.7)	25.6 (7.8)	9 (47.4)	3.67	7
Bawazeer et al. (2010)	Saudi Arabia	Asia	Retrospective study	ADA	11	11 (100.0)	28.4 (5.6)	10 (90.9)	0.9	4
Bejerano et al. (2013)	Spain	Europe	Retrospective study	IFX, ADA	63	36 (57.1)	38.9 (9.0)		2	5
Calvo-Río et al. (2013)	Spain	Europe	Retrospective study	IFX, ADA	108	59 (54.6)	38.2 (10.4)		2	6
Calvo-Rio et al. (2014)	Spain	Europe	Retrospective study	IFX, ADA	124	68 (54.8)	38.6 (10.4)	84 (67.7)	1	8
Cantini et al. (2012)	Italy	Europe	Prospective study	IFX	50	20 (40.0)	37.5 (12.3)	31 (62.0)	2	8
Yalcindag and Kose (2019)	Turkey	Asia	Retrospective study	IFX	20	13(65.0)	27.9(4.2)	16 (80.0)	1.73	4
Domínguez Casas et al. (2017)	Spain	Europe	Retrospective study	IFX	100	54 (54.0)	40.7 (10.1)		1	4
El Garf et al. (2018)	Egypt	Africa	Prospective study	IFX	20	17 (85.0)	31.8 (9.1)	14 (70.0)	1.17	6
Fabiani et al. (2017)	Italy	Europe	Retrospective study	ADA	40	22 (55.0)	41.9 (12.0)	21 (95.5)	1	8
Hamuryudan et al. (2013)	Turkey	Asia	Retrospective study	IFX	43	33 (76.7)	31.0 (8.4)	11 (34.4)	2.42	5
Interlandi et al. (2014)	Italy	Europe	Retrospective study	ADA	12	11 (91.7)	24.3 (8.6)	11 (91.7)	1.75	7
Katsuyama et al. (2019)	Japan	Asia	Retrospective study	IFX	11	8 (72.7)	45.1 (8.6)		2	7
Keino et al. (2011)	Japan	Asia	Retrospective study	IFX	14	12 (85.7)	38.0	8 (57.1)	1.58	7
Martín-Varillas et al. (2018)	Spain	Europe	Retrospective study	ADA	74	39 (52.7)	38.7 (11.3)	65 (87.8)	2.89	8
Mesquida et al. (2013)	Spain	Europe	Retrospective study	IFX, ADA	32	16 (50.0)	39.0 (11.9)	28 (87.5)	6.44	6
Okada et al. (2012)	Japan	Asia	Prospective study	IFX	63	56 (88.9)	37.4 (11.6)	21 (43.8)	1	5
Takeuchi et al. (2014)	Japan	Asia	Retrospective study	IFX	164	133 (81.1)	40.0 (11.7)	67 (40.9)	2.74	8

NA, not available; ADA, adalimumab; IFX, infliximab.

a The number of patients with ocular remission (the proportion of patients with ocular remission).

b The mean duration of follow-up.

c Study quality was assessed using the Newcastle-Ottawa Scale. The score indicates the percentage of items met by each included study.

### Meta-Analysis and Subgroup Analysis

The results of the meta-analysis and subgroup analysis were demonstrated in **Table 2**. The pooled results included the rate of inflammation remission, VA improvement, CMT decrease, and CS-sparing effects. Heterogeneity was high in the analysis of inflammation remission (*I^2^ = *89.3%), but was not detected in the subgroups of prospective study group (*I^2^ = *59.2%) and ADA therapy group (*I^2^ = *0.0%). Similarly, heterogeneity was high in the analysis of VA improvement (*I^2^ = *86.6%), but was not detected in the subgroups of Europe (*I^2^ = *0.0%), prospective study group (*I^2^ = *0.0%), and ADA therapy group (*I^2^ = *22.2%). The subgroup analysis indicated the study location, study design and the kind of anti-TNF-α agent contributed to such heterogeneity exited.

**Table 2:** The results of the meta-analysis and subgroup analysis.

		No. of studies	Pooled effect sizes (95% CI)	Heterogeneity		*P* of Egger’s for publication bias
				*I^2^* (%)	*P*	
**Inflammation remission (%)**		15	68.2 (59.1, 78.8)	89.3	<0.001	0.014
Study location						
	Asia	7	54.3 (39.7, 74.4)	88.5	<0.001	
	Europe	6	82.1 (72.6, 92.7)	82.9	<0.001	
Study design						
	Retrospective study	11	71.3 (60.9, 83.5)	90.2	<0.001	
	Prospective study	3	58.3 (45.6, 74.6)	59.2	0.129	
Follow up (months)						
	<24	8	75.4 (64.2, 88.4)	83.1	<0.001	
	≥24	6	58.7 (43.7, 78.8)	93.5	<0.001	
Anti TNF agents						
	IFX	8	53.7 (43.1, 66.8)	77.6	<0.001	
	ADA	4	91.3 (86.4, 96.5)	0.0	0.639	
	IFX, ADA	2	76.9 (59.8, 98.8)	87.3	<0.001	
**Corticosteroid suspended**		5	38.4 (22.8, 64.6)	85.9	<0.001	0.116
Study location						
	Asia	2	44.4 (16.1, 100.0)	94.2	<0.001	
	Europe	3	34.3 (17.8, 66.0)	76.0	<0.001	
Follow up (months)						
	<24	2	30.5 (7.9, 100.0)	87.9	<0.001	
	≥24	3	42.3 (21.8, 82.1)	89.6	<0.001	
Anti TNF agents						
	IFX	2	44.4 (16.1, 100.0)	94.2	<0.001	
	ADA	2	30.5 (7.9, 100.0)	87.9	<0.001	
**Corticosteroid tapered**		3	33.9 (16.4, 69.8)	71.8	0.029	0.210
**CMT decrease(μm)** ^**a**^		5	-112.7 (-152.6, -72.8)	70.2	0.009	0.911
Follow up (months)						
	<24	2	-107.8 (-187.7, -27.8)	87.0	<0.001	
	≥24	3	-117.1 (-169.0, -65.2)	61.5	0.021	
Anti TNF agents						
	IFX	2	-102.1 (-177.0, -27.2)	76.8	<0.001	
	IFX, ADA	3	-121.1 (-170.7, -71.4)	67.2	0.011	
**VA improvement**		8	60.3 (47.2, 77.0)	86.6	<0.001	0.206
Study location						
	Asia	5	44.1 (31.0, 62.7)	72.6	0.054	
	Europe	2	86.8 (77.7, 96.9)	0.0	0.391	
Study design						
	Retrospective study	6	50.7 (35.4, 72.6)	88.0	<0.001	
	Prospective study	2	82.3 (72.9, 92.9)	0.0	0.784	
Anti TNF agents						
	IFX	6	52.5 (38.5, 71.4)	87.6	<0.001	
	ADA	2	86.5 (71.0, 100.0)	22.2	0.186	
Follow up (months)						
	<24	4	76.2 (59.5, 97.7)	63.8	0.015	
	≥24	4	49.3 (33.0, 73.6)	90.9	<0.001	

ADA, adalimumab; IFX, infliximab; CS, corticosteroid; CMT, central macular thickness; VA, visual acuity.

a Difference between before and after treatment.

In addition, the presence of publication bias was not detected across the studies reporting (P > 0.1) other than in the analysis of inflammation remission (P=0.014). The funnel plots were demonstrated in **Supplementary Figure 1**, and the P values of Egger’s test were list in **Table 2**.

### Inflammation Remission

**Figure 2:**
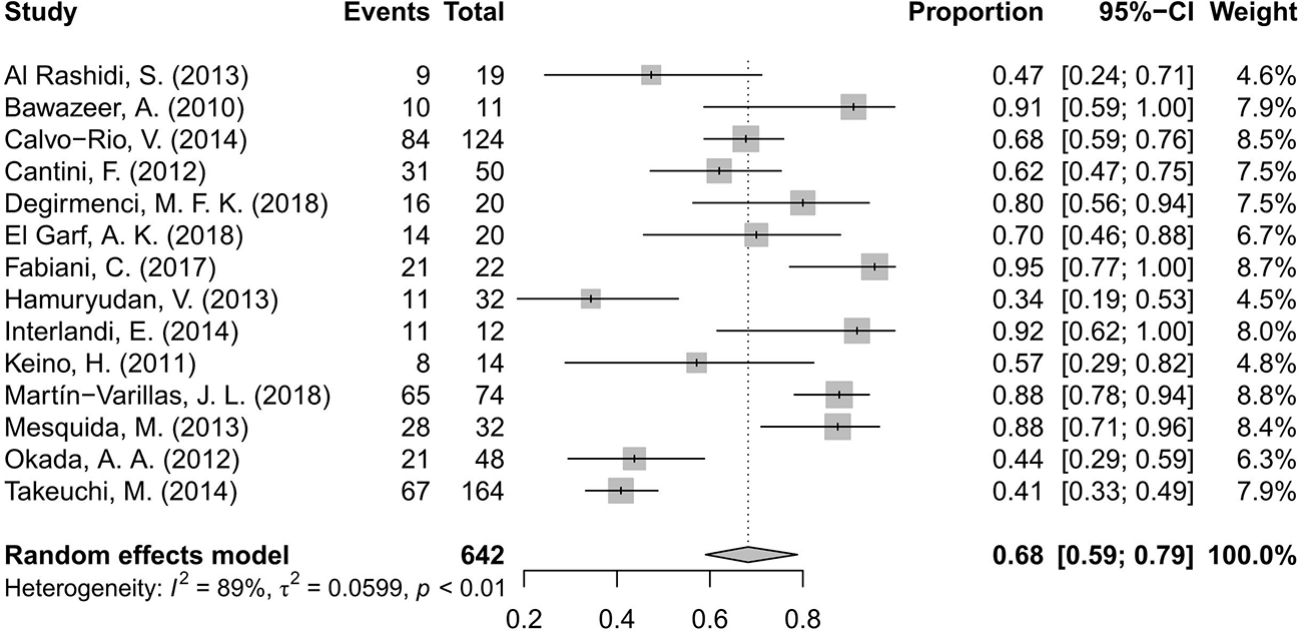
Forest plot of the inflammation remission rate after anti-tumor necrosis factor-alpha (TNF-α) treatment.

Fourteen studies (Bawazeer et al., 2010; Keino et al., 2011; Cantini et al., 2012; Okada et al., 2012; Al Rashidi et al., 2013; Hamuryudan et al., 2013; Mesquida et al., 2013; Calvo-Río et al., 2014; Interlandi et al., 2014; Takeuchi et al., 2014; Fabiani et al., 2017; El Garf et al., 2018; Martín-Varillas et al., 2018; Yalcindag and Kose, 2019) including 642 patients of BD-associated uveitis were evaluated for the rate of inflammation remission after anti-TNF-α therapy. The total number of included patients and the number who reached inflammation remission during the follow-up time in each study were retrieved. A random effects model was used for the test of heterogeneity (89%, p<0.01). The overall pooled remission rate was 68%, with a 95% CI of 0.59–0.79 (**Figure 2**). The forest plot showed that the diamond marker did not intersect 1, suggesting the anti-TNF-α therapy reached a 68% inflammation remission rate with significant difference.

### Visual Acuity Improvement

**Figure 3:**
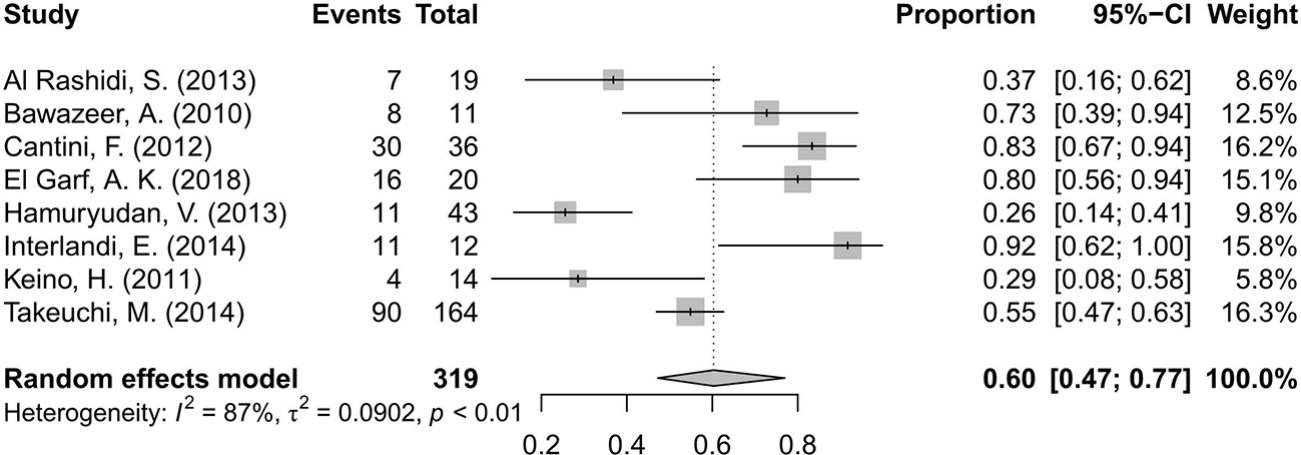
Forest plot of visual acuity (VA) improvement after anti-TNF-α treatment.

A total of eight studies (Bawazeer et al., 2010; Keino et al., 2011; Cantini et al., 2012; Al Rashidi et al., 2013; Hamuryudan et al., 2013; Interlandi et al., 2014; Takeuchi et al., 2014; El Garf et al., 2018) involving 319 patients presented the number of patients with improvement in VA after the intervention. The overall pooled proportion of patients with improved VA was 60%, with a 95% CI of 0.47–0.77. The test of heterogeneity was 87% (p<0.01), suggesting that a random-effects model was preferred. The forest plot showed that the diamond marker did not intersect 1, suggesting that the 60% pooled prevalence was significantly higher than 0 (**Figure 3**).

Bejerano, C. (Bejerano et al., 2013) reported that VA improved from 0.5 ± 0.3 at baseline to 0.7 ± 0.3 after 2 years (p<0.001) in 63 patients/110 affected eyes. El Garf, A. K. (El Garf et al., 2018) presented 36 affected eyes (16 bilateral and 4 unilateral) in which VA significantly improved (p <0.05) from 0.1 ± 0.09 at baseline to 0.6 ± 0.2 at week 8 and 0.8 ± 0.2 at week 32, respectively. There was no additional improvement at week 56, when the VA was still 0.8 ± 0.2. In Takeuchi’s study (Takeuchi et al., 2014) in 2014, the mean best corrected visual acuity (BCVA) was recorded as the logarithm of the minimum angle of resolution (logMAR). The VA significantly improved when the duration of infliximab treatment ranged from 12 to 48 months (P < 0.05), but did not improve when the patients needed more than 48 months treatment. Because the values of VA were recorded in different methods, like the decimal vision and logMAR vision, a meta-analysis on the amount of VA improvement was not conducted.

### Central Macular Thickness Decrease

**Figure 4:**
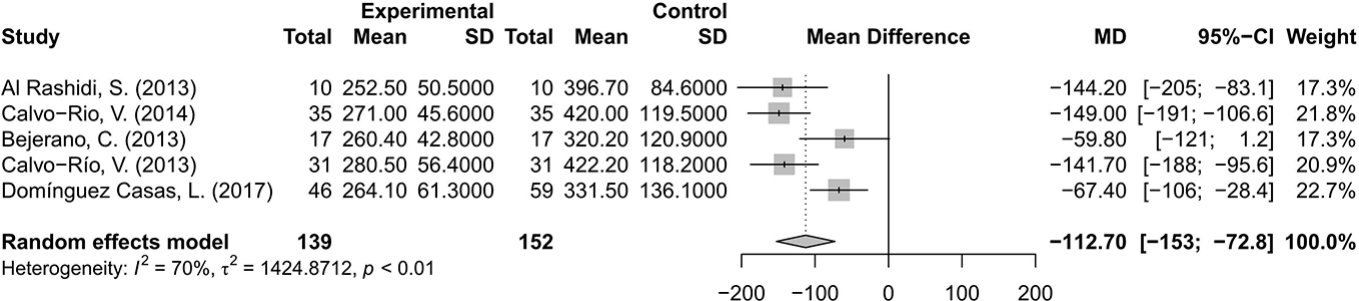
Forest plot of central macular thickness (CMT) reduction after anti-TNF-α treatment.

The changes of CMT were evaluated and analyzed in five studies (Al Rashidi et al., 2013; Bejerano et al., 2013; Calvo-Río et al., 2013; Calvo-Río et al., 2014; Domínguez Casas et al., 2017). The pooled results suggested that CMT decreased by 112.70 μm, with a 95% CI of 72.8–153 μm, indicating significant difference. A random effects model was used for the test of heterogeneity (70%, p<0.01) (**Figure 4**).

### Corticosteroid-Sparing Effects

**Figure 5:**
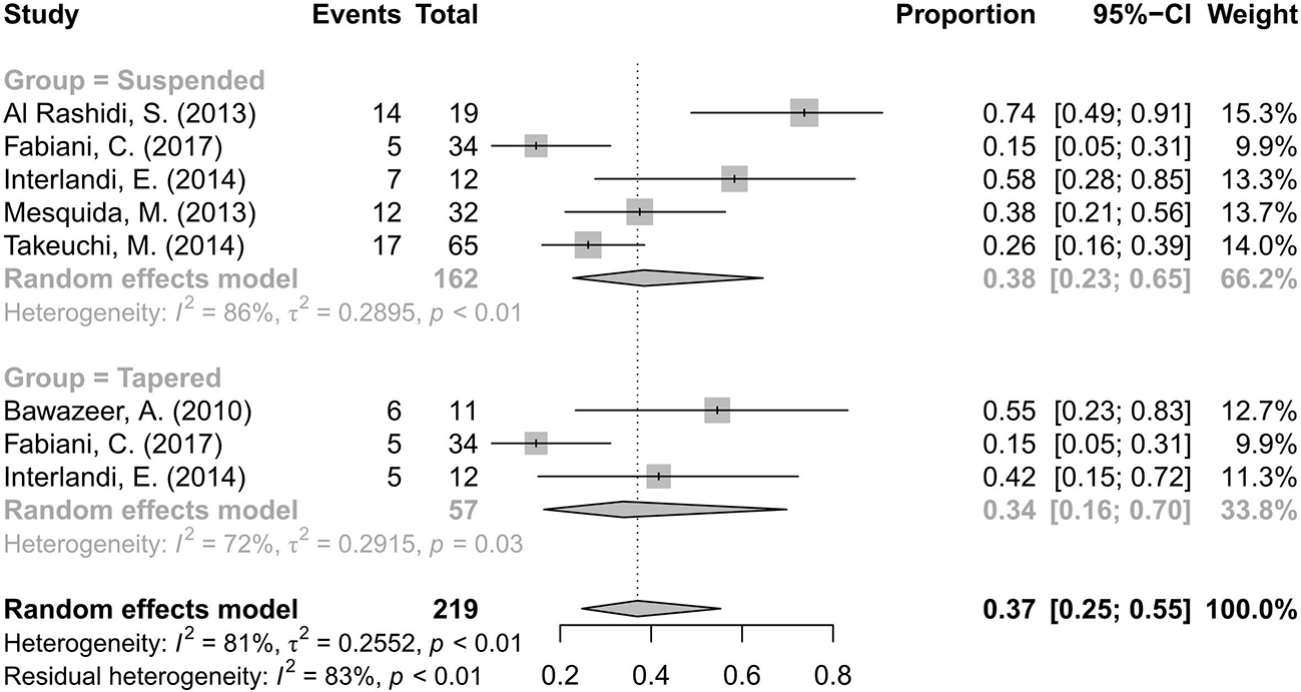
Forest plot of the corticosteroid (CS)-sparing effects of anti-TNF-α treatment. The analysis was conducted by dividing studies into “suspended” and “tapered” according to changes in the CS dosage.

Information on the CS-sparing were extracted and analyzed. Fifty-five (33.95%) of 162 patients completely discontinued CS in five articles (Al Rashidi et al., 2013; Mesquida et al., 2013; Interlandi et al., 2014; Takeuchi et al., 2014; Fabiani et al., 2017), and 16 (28.07%) of 57 patients had a CS dose reduction in three articles (Bawazeer et al., 2010; Interlandi et al., 2014; Fabiani et al., 2017). The pooled proportions of CS-suspended and CS-tapered patients were 38% with a 95% CI of 0.23–0.65 and 34% with a 95% CI of 0.16–0.70, respectively. The differences were statistically significant because the diamond markers did not intersect 1 in the forest plot (**Figure 5**).

Only two studies mentioned the dosage in detail. The mean daily dose of CS decreased from 26.87 to 3.33 mg/day (p=0.002) in Interlandi E. (Interlandi et al., 2014) and from 18.25 mg (range 12.5–17.5 mg) to 7.9 mg (range 5–10 mg) (P<0.05) in Bawazeer A. (Bawazeer et al., 2010) during anti-TNF-α therapy.

### Adverse Events

A total of 14 studies (Keino et al., 2011; Al Rashidi et al., 2013; Bejerano et al., 2013; Calvo-Río et al., 2013; Hamuryudan et al., 2013; Mesquida et al., 2013; Calvo-Río et al., 2014; Takeuchi et al., 2014; Domínguez Casas et al., 2017; Fabiani et al., 2017; El Garf et al., 2018; Martín-Varillas et al., 2018; Katsuyama et al., 2019; Yalcindag and Kose, 2019) included safety information with more than 109 patients (**Table 3**). According to the available data, 22 patients (2.62%) experienced severe AEs. The result of incidence rate excluded two studies in which information on severe AEs was not available. The severe AEs included severe infusion reactions (n=3), melanoma (n=1), lymphoma (n=3), pneumonia (n=6), bacteremia (n=3), and tuberculosis (n=6). The most frequently reported mild AEs were infusion reactions (rash, itchiness, or respiratory distress), upper respiratory inflammation, gastroenteritis, and urinary tract infections.

**Table 3:** Adverse events reported in the included studies.

First author (year)	Sample size (n)	Follow-up (years)^a^	AEs (n)	Severe AEs (%)^b^	Detailed safety information
Al Rashidi et al. (2013)	19	3.67	1	1 (5.26)	One patient developed a severe infusion reaction during the 19th scheduled infusion
Bawazeer et al. (2010)	11	0.9	0	0 (0)	None
Bejerano et al. (2013)	63	2	1	1 (1.59)	Miliary TB in 1 patient at month 1 after the onset of IFX.
Calvo-Río et al. (2013)	108	2	NA	NA	NA
Calvo-Rio et al. (2014)	124	1	NA	3 (2.4)	Minor side effects: mild infusion reactions and local reactions (pain and erythema). Severe AEs were observed in 3 case: one patient had miliary TB after 1 month of IFX, another was diagnosed with non-Hodgkin’s lymphoma in month 6 of ADA treatment, and one patient who died was diagnosed with melanoma in month 3 of treatment with ADA.
Cantini et al. (2012)	50	2	0	0 (0)	None
Yalcindag and Kose (2019)	20	1.73	3	NA	Three patients were switched to a different agent because of AEs.
Domínguez Casas et al. (2017)	100	1	4	4 (4)	Severe AEs were lymphoma (n=1), pneumonia (1), and bacteriemia (2 by *Escherichia coli*)
El Garf et al. (2018)	20	1.17	8	0 (0)	Mild infusion reactions in 4 patients, frequent urinary tract infections in 1 patient, and frequent upper airway infections in 3 patients
Fabiani et al. (2017)	40	1	1	1 (2.27)	One severe AE: pneumonia.
Hamuryudan et al. (2013)	43	2.42	5	4 (9.30)	Pulmonary TB in 3 patients (in month 17, 32, and 46 of IFX treatment). Pneumonia in 1 patient and depression in 1 patient.
Interlandi et al. (2014)	12	1.75	0	0 (0)	None
Katsuyama et al. (2019)	11	2	4	0 (0)	Urinary tract infection in 1 patient and significant cataract progression in 1 patient. Two patients exhibited transient elevation of serum creatinine levels.
Keino et al. (2011)	14	1.58	10	0 (0)	Infusion reactions (rash, itchiness and/or respiratory distress) in 4 patients and common cold symptoms in 5 patients. One patient had suspected bacterial pharyngitis that responded to antibiotic therapy.
Martín-Varillas et al. (2018)	74	2.89	4	4 (5.41)	Severe AEs were found in 4 patients (lymphoma, pneumonia, severe local reaction at the injection site, and bacteremia by *Escherichia coli*; 1 each).
Mesquida et al. (2013)	32	6.44	3	2(5.25)	One severe infusion reaction, 1 case of pulmonary tuberculosis, and 1 case of prostatitis
Okada et al. (2012)	63	1	NA	0(0)	Urticaria and rash, infusion reactions and decreased blood pressure were observed, but the number with each AE was not mentioned.
Takeuchi et al. (2014)	164	2.74	65	2 (1.22)	AEs were observed in 65 cases and included infusion reaction (21% incidence), upper respiratory inflammation (4% incidence) and gastroenteritis (3% incidence). Pneumonia occurred in 2 patients (3%).^c^

NA, not available; AEs, adverse events; IFX, infliximab; ADA, adalimumab; TB, tuberculosis.

a The mean duration of follow-up.

b The percentage of severe AEs observed in all subjects in each study, marked as a number (percentage).

c The incidence indicates the proportion of patients with one particular reaction out of all of the AEs reported in this study.

## Discussion

TNF-α is a pro-inflammatory cytokine produced by Th1 lymphocytes and macrophages and is expressed at a significantly higher level in BD patients compared to in healthy controls (De Vos et al., 1992; Santos Lacomba et al., 2001). It has been reported that TNF-α plays a key role in the development and persistence of uveitis in BD (Lim et al., 2007). Therefore, anti-TNF-α therapy may provide good efficacy in BD-associated uveitis (Fleisher et al., 1990; Sartani et al., 1996; Sfikakis et al., 2004). In this meta-analysis, we summarized the available evidence on the use of anti-TNF-α in the treatment of BD-associated uveitis.

We presented a pooled proportion for the inflammation remission rate of 68%, which was lower than the results presented in other reviews. Shuai Ming et al. (Ming et al., 2018) presented that the proportions of activity controlled were 74% and 79% in non-infectious uveitis with adalimumab at ≤6 and ≥12 months follow-up respectively. The slightly lower rate obtained in our review may due to that the refractory of BD-associated uveitis than other cases. Furthermore, Simonini et al (Simonini et al., 2014) summarized the inflammation remission rate of anti-TNF-α treatment in childhood BD-associated uveitis with a pooled proportions of 87% in the ADA group and 72% in the IFX group, which were both higher than our data. In consideration of the patients’ age of our review (24.3–45.1 years), the children could have better inflammation control than adults. Some retrospective (Fujikawa and Suemitsu, 1997; Treudler et al., 1999) surveys had shown that ocular complications and, notably, blindness were less frequent in the children-onset group than in the adult group. According this review and analysis it was concluded that BD-associated ocular inflammation can be effectively controlled by anti-TNF-α agents.

This review indicated the pooled rate of VA improvement was 60%. There was a point we should indicated that this rate did not include patients with stable vision. Hence, the proportion of patients with VA preservation (stable VA and improved VA) should be higher, as was found in Shuai Ming’s analysis (Ming et al., 2018). In his analysis, ADA effectively preserved VA in 88.8% of the patients.

The decrease of CMT was as much as 112.70 μm by anti-TNF-α agents invention, which indicated that both structure and function of macular could therefore be substantially improved. The anti-TNF-α treatment had prominent efficacy in refractory CME related to BD as reported (Al Rashidi et al., 2013; Calvo-Río et al., 2014). Markomichelakis NN (Markomichelakis et al., 2004) conducted a study on the efficacy of infliximab for treatment of chronic CME due to uveitis. He included 10 patients with a long-standing CME but without active inflammation at the time of infliximab infusion. The macular thickness was reduced from 428 ± 138 μm to 219 ± 51 μm at 2 months. It suggested that TNF-α might play a pathogenetic role in chronic CME, and the mechanism of improving CME of anti-TNF-α might be not directly related to inflammation.

We categorized the outcome of CS-sparing effects into two subtypes: the proportion of patients of CS-suspended and of CS-tapered. In this analysis, 38% of patients were CS-suspended and 34% were CS-tapered. The mean daily dose of CS decreased from 26.87 to 3.33 mg/day in the report of Interlandi E. (Interlandi et al., 2014) and from 18.25 to 7.9 mg in the report of Bawazeer A. (Bawazeer et al., 2010). We could infer from these findings that many patients obtained benefit from the CS-sparing effects of the anti-TNF-α treatment, which was associated with not only better outcomes and but also better tolerability.

The results mentioned above suggested that anti-TNF-α provided satisfactory control of ocular inflammation, improved visual prognosis and allowed good preservation of macular structural integrity. It was consistent with the results in a meta-analysis by Urruticoechea-Arana A et al. (Urruticoechea-Arana et al., 2018). He presented that patients treated with anti-TNF-α agents had a lower risk of uveitic flare or visual impairment over the long-term than those in conventional therapy group. What’s more, treatment with anti-TNF-α drugs yielded a significant CS-sparing effect, which reduced the occurrence of steroid-related complications such as glaucoma, cataract, obesity, hirsutism, gastrohelcosis, and growth retardation. It seemed that anti-TNF-α agents would have positive effects on long-term prognosis for BD-associated uveitis.

However, it was necessary to address the safety of anti-TNF-α treatment here. In this review, we found that 22 patients (2.62% of all included patients) experienced severe AEs (Al Rashidi et al., 2013; Bejerano et al., 2013; Hamuryudan et al., 2013; Mesquida et al., 2013; Calvo-Río et al., 2014; Domínguez Casas et al., 2017; Fabiani et al., 2017; Martín-Varillas et al., 2018), while some minor AEs such as infusion reactions (rash, itchiness, or respiratory distress) and gastroenteritis happened frequently. Infectious AEs, such as tuberculosis, pneumonia, bacteremia, urinary tract, and upper airway infections, were the main severe AEs. These may result from the suppression of the immune system by anti-TNF-α treatment, so we should be alert to signals of early infection so that in-time intervention cloud be implemented, especially for elder patients. In addition, melanoma and lymphoma were reported in the review, so ophthalmologists should be aware of these rare but fatal AEs. Better understanding of the drug-related complications calls for studies with long-term duration of follow-up.

## Conclusions

In conclusion, by reviewing and analyzing the 18 articles, the results presented that anti-TNF-α treating was one of effective therapeutic method for BD-associated uveitis. However, clinicians should pay more attention to prevent patients from having severe AEs, especially for elder patients. Fortunately, the incidence of severe AEs was not very high.

### Limitation

Several limitations of our analyses must be mentioned. First of all, there were few RCT articles for us to review and only 3/18 articles were prospective uncontrolled research. Differences in follow-up durations and observation endpoints across different trials may have affected the results. Secondly, high heterogeneity was observed in our analysis. We found that study location, study design, and the kind of anti-TNF-α agents (ADA or IFX) contributed to the heterogeneities. The presence of publication bias detected in the analysis of inflammation remission was also a limitation in our analysis (**Table 2** and **Supplementary Figure 1**). It is an urgent for more high-quality and large-scale RCTs to better evaluate the efficacy and safety profile of anti-TNF-α treatment in patients with BD-associated uveitis.

## Author Contributions 

Conception and design: WS and DL. Drafting and revising of the article: YH, ZH, SY, and XC. Final approval: WS and DL.

## Funding

Supported by grants from National Natural Science Foundation of China (U1601226, 81870649, and 81670897); Guangdong Natural Science Funds for Distinguished Young Scholar (2016A030306006).

## Conflict of Interest

The authors declare that the research was conducted in the absence of any commercial or financial relationships that could be construed as a potential conflict of interest.

## Footnotes

1
https://www.accessdata.fda.gov/scripts/cder/daf/index.cfm?event=overview.process&ApplNo=125057

2
https://news.abbvie.com/alert-topics/immunology/abbvies-humira-adalimumab-receives-chmp-positive-opinion-to-treat-certain-forms-non-infectious-uveitis-disease-that-can-severely-impact-vision1.htm

## Supplementary Material

Supplementary Figure 1 Funnel plots for publication bias using Egger’s text. The Egger’s test of A) the analysis of inflammation remission rate, B) the analysis of corticosteroid-suspended rate, C) the analysis of corticosteroid-tapered rate, D) the analysis of central macular thickness decrease and E) the analysis of improved visual acuity.
