# Supplementary figures and images for: Effectiveness and Safety of Anti-Tumor Necrosis Factor-Alpha Agents Treatment in Behcets’ Disease-Associated Uveitis: A Systematic Review and Meta-Analysis
Source: Front Pharmacol. 2020 Jun 24;11:941. doi: 10.3389/fphar.2020.00941 (PMC7327708; doi:10.3389/fphar.2020.00941)

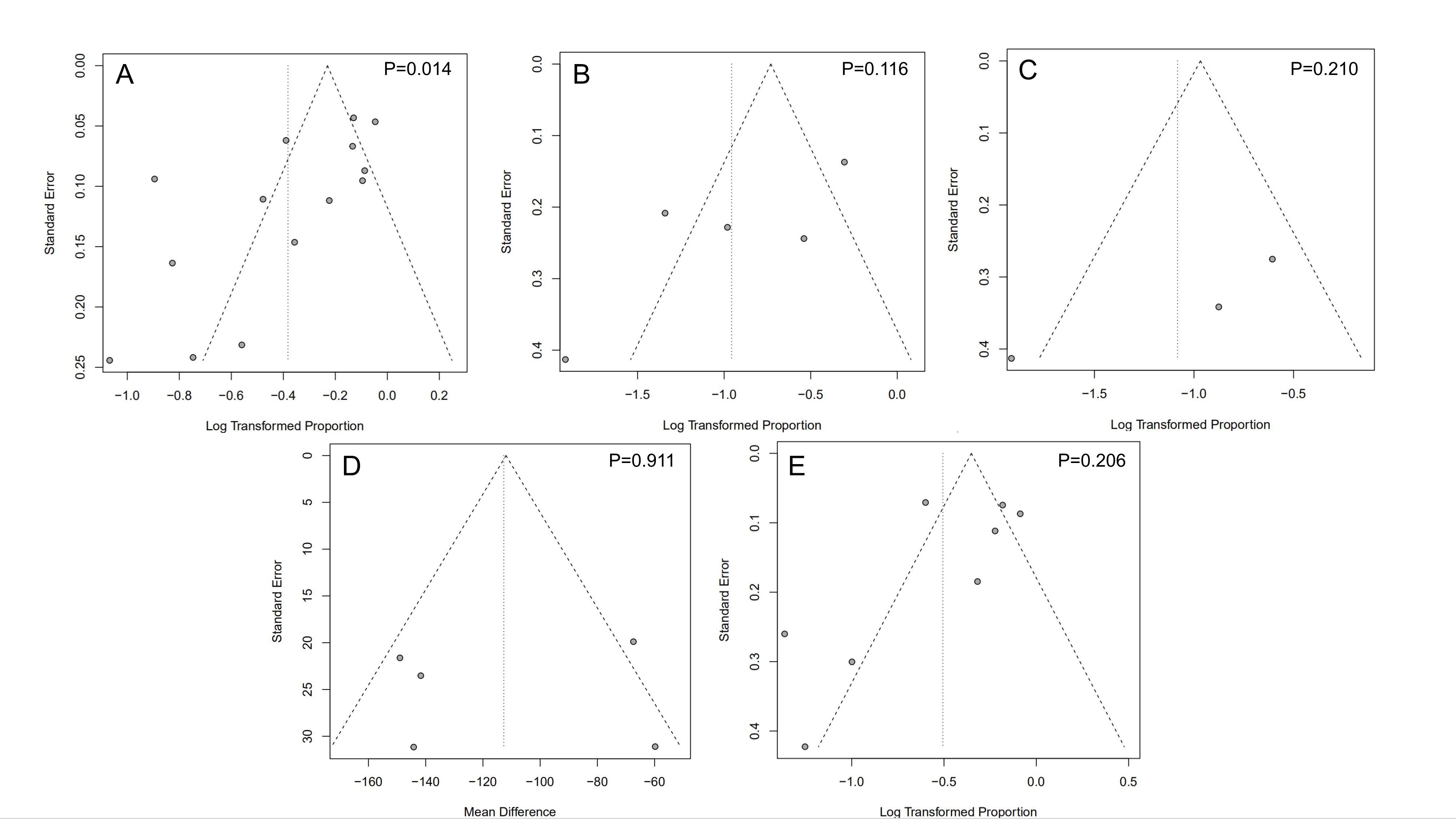

Supplement: Supplementary Figure 1 — Funnel plots for publication bias using Egger’s text. The Egger’s test of A) the analysis of inflammation remission rate, B) the analysis of corticosteroid-suspended rate, C) the analysis of corticosteroid-tapered rate, D) the analysis of central macular thickness decrease and E) the analysis of improved visual acuity. [file Image_1.jpeg]
