# Supplementary material for: Effectiveness and Safety of Anti-Tumor Necrosis Factor-Alpha Agents Treatment in Behcets’ Disease-Associated Uveitis: A Systematic Review and Meta-Analysis
Source: Front Pharmacol. 2020 Jun 24;11:941. doi: 10.3389/fphar.2020.00941 (PMC7327708; doi:10.3389/fphar.2020.00941)
Supplement: Supplementary file 2 [file Table_1.docx]

**Supplementary Table 1: Expanded information of the included studies**

| First author (year) | Study design | Data source and patients included | Search query (DOI/PMID/PUI) |
| --- | --- | --- | --- |
| Al Rashidi (2013) | Retrospective study  Case series | Records of patients with refractory visionthreatening uveitis associated with BD treated with IFX at King Abdulaziz University Hospital, Riyadh were retrospectively reviewed | 10.3109/09273948.2013.779727 |
| Bawazeer (2010) | Retrospective study  Case series | Records of the patients with ocular BD received ADA treatment between June 2007 and May 2009 were collected in Maghrabi Eye Hospital, a tertiary referral hospital in Jeddah, Saudi Arabia. | 10.3109/09273948.2010.483314 |
| Bejerano (2013) | Retrospective study  Case series | Multicentre study of patients followed in the Uveitis Clinic of 24 hospitals. All of them were refractory to conventional therapy and treated with ADA or IFX. | 10.1136/annrheumdis-2012-eular.2670 |
| Calvo-Río (2013) | Retrospective study  Case series | Multicentre study of BD-assciated uveitis patients from 32 hospitals. All of them presented inadequate response to standard IS and switched to ADA or IFX. | 10.1136/annrheumdis-2013-eular.1874 |
| Calvo-Rio (2014) | Retrospective study  Case series | Multicentre study of BD uveitis refractory to IS. Patients were treated with ADA or IFX consequently. | 10.1093/rheumatology/keu266 |
| Cantini (2012) | Prospective study  Uncontrolled pilot study | Single center, prospective, 6-year duration, follow-up study on patients with refractory BD-associated posterior uveitis. The patients received IFX therapy. | 10.2147/BTT.S27343 |
| Yalçindag (2019) | Retrospective study  Cohort study | Medical records of patients with BD-associated uveitis treated with IFX or IFNα-2a between January 2010 and April 2018, were retrospectively analyzed. | 10.1080/09273948.2019.1606256 |
| Domínguez Casas (2017) | Retrospective study  Case series | Multicentre study of patients with BD-associated uveitis refractory to conventional IS drug and switched to IFX therapy. | 10.1136/annrheumdis-2017-eular.6226 |
| El Garf (2018) | Prospective study  Uncontrolled pilot study | Patients with refractory Behcet's posterior uveitis received IFX therapy at the dose of 5 mg/kg at weeks 0, 2, 6 (induction) and every 8 weeks for a maximum of 6 infusions. | 10.1016/j.ejr.2017.08.001 |
| Fabiani (2017) | Retrospective study  Case series | Multicentre study, retrospectively collected medical documentation from patients suffering from BDrelated uveitis resistant to conventional IS therapy and consequently treated with ADA. | 10.1007/s10067-016-3480-x |
| Hamuryudan (2013) | Retrospective study  Case series | The charts of BD-associated patients treated with IFX were reviewed retrospectively. | 10.1002/art.38216 |
| Interlandi (2014) | Retrospective study  Case series | Singe centre study of uveitis patients affected by BD, treated with ADA between 2008 and 2012. Their data were selected from archives and analysed. | 25005224(PMID) |
| Katsuyama (2019) | Retrospective study  Case series | Medical records of patients with BD uveoretinitis refractory to conventional IS treatment who had been treated with IFX therapy. | 10.2147/opth.s198648 |
| Keino (2011) | Retrospective study  Case series | Medical records were reviewed of patients with ocular BD followed in the Ocular Inflammation Service at the Kyorin Eye Center, Kyorin University Hospital (Tokyo, Japan) who received IFX therapy. | 10.1136/bjo.2010.194464 |
| Martín-Varillas (2018) | Retrospective study  Case series | Open-label multicenter study of ADA-treated patients with BD-associated uveitis refractory to IS drugs. | 10.1016/j.ophtha.2018.02.020 |
| Mesquida (2013) | Retrospective study  Case series | Retrospective study of patients with Behcet's uveitis treated with IFX or ADA in four tertiary referral hospitals of Spain. | PUI: L628730830 |
| Okada (2012) | Prospective study  Uncontrolled pilot study | Data were collected prospectively at 8 tertiary uveitis centers of BD-associated uveitis who were refractory to conventional treatments and switched to IFX. | 10.1001/archophthalmol.2011.2698 |
| Takeuchi (2014) | Retrospective study  Case series | Multicenter retrospective study using questionnaires of patients with BD-associated uveitis treated with IFX for more than 1 year. | 10.1016/j.ophtha.2014.04.042 |

Abbreviations: Behcet disease, BD; ADA, Adalimumab; IFX, Infliximab; immunosuppressants, IS.
